# 2,6-Bis(trifluoro­meth­yl)benzoic acid

**DOI:** 10.1107/S1600536809016468

**Published:** 2009-05-07

**Authors:** John M. Tobin, Jason D. Masuda

**Affiliations:** a Department of Chemistry, Saint Mary’s University, 923 Robie Street, Halifax, NS, Canada B3H 3C3

## Abstract

The title compound, C_9_H_4_F_6_O_2_, contains two mol­ecules in the asymmetric unit, one of which exhibits disorder in both of its trifluoro­methyl groups. The dihedral angles between the benzene ring and the carboxyl group are 71.5 (2) and 99.3 (2)° in the two independent mol­ecules. The compound exhibits a catemeric structure resulting from inter­molecular O—H⋯O hydrogen bonding between the carboxyl groups.

## Related literature

There is only one example in the literature of a crystallographically characterized benzoic acid with trifluoro­methyl groups in the *ortho* position, namely 2-trifluoro­methyl-3-pyrrole benzoic acid (see Faigl *et al.*, 1999 ▶). For a recent example of crystal engineering to promote the formation of dimeric or catemeric structures in benzoic acids, see: Moorthy *et al.* (2002 ▶). For synthesis details, see: Dmowski & Piasecka-Macieiewska (1998 ▶).
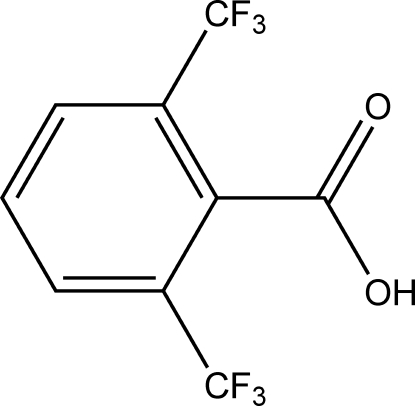


## Experimental

### 

#### Crystal data

C_9_H_4_F_6_O_2_
*M* *_r_* = 258.12
Monoclinic, 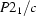

*a* = 10.873 (2) Å
*b* = 15.755 (3) Å
*c* = 11.561 (2) Å
β = 94.961 (2)°
*V* = 1973.0 (6) Å^3^
*Z* = 8
Mo *K*α radiation
μ = 0.20 mm^−1^
*T* = 296 K
0.39 × 0.31 × 0.26 mm

#### Data collection

Bruker APEXII CCD diffractometer
Absorption correction: multi-scan (*SADABS*; Bruker, 2008 ▶) *T*_min_ = 0.834, *T*_max_ = 0.951
12904 measured reflections
3438 independent reflections
2889 reflections with *I* > 2σ(*I*)
*R*_int_ = 0.021

#### Refinement

*R*[*F* ^2^ > 2σ(*F* ^2^)] = 0.039
*wR*(*F* ^2^) = 0.112
*S* = 1.02
3438 reflections
331 parameters
H-atom parameters constrained
Δρ_max_ = 0.21 e Å^−3^
Δρ_min_ = −0.23 e Å^−3^

### 

Data collection: *APEX2* (Bruker, 2008 ▶); cell refinement: *SAINT* (Bruker, 2008 ▶); data reduction: *SAINT*; program(s) used to solve structure: *SHELXS97* (Sheldrick, 2008 ▶); program(s) used to refine structure: *SHELXL97* (Sheldrick, 2008 ▶); molecular graphics: *ORTEP-3 for Windows* (Farrugia, 1997 ▶) and *Mercury* (Macrae *et al.*, 2006 ▶); software used to prepare material for publication: *SHELXTL* (Sheldrick, 2008 ▶).

## Supplementary Material

Crystal structure: contains datablocks I, global. DOI: 10.1107/S1600536809016468/bi2369sup1.cif

Structure factors: contains datablocks I. DOI: 10.1107/S1600536809016468/bi2369Isup2.hkl

Additional supplementary materials:  crystallographic information; 3D view; checkCIF report

## Figures and Tables

**Table 1:** Hydrogen-bond geometry (Å, °)

*D*—H⋯*A*	*D*—H	H⋯*A*	*D*⋯*A*	*D*—H⋯*A*
O2—H2*A*⋯O4^i^	0.82	1.82	2.6340 (19)	169
O3—H3*A*⋯O1	0.82	1.88	2.6951 (18)	176

Symmetry code: (i) 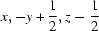.

**Fig. 1.**
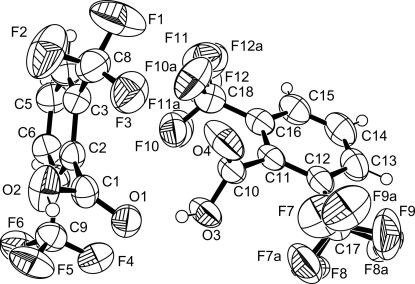
The two molecules in the asymmetric unit with displacement ellipsoids shown at 50% probability for non-H atoms. For the disordered CF3 groups, both disorder components are shown.

**Fig. 2.**
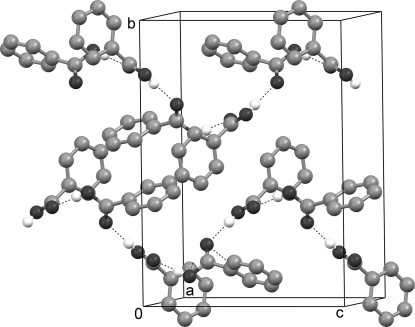
Ball and stick representation featuring the catemeric structure formed through O—H···O hydrogen bonding. H atoms not involved in H-bonding and the CF3 groups have been omitted for clarity.

## supplementary crystallographic information

### Comment

The title molecule crystallizes in a catemer motif, a relatively rare form compared to the typical dimeric motif exhibited by benzoic acids resulting from intermolecular hydrogen bonding between the carboxylic acid groups (Moorthy *et al.*, 2002). The sterically bulky *o*-CF_3_ groups result in the carboxylic acid fragments being twisted with respect to the aryl ring. This results in dihedral angles between the aryl ring and carboxylic acid fragments of C7—C2—C1—O1 = 71.5 (2)° and C12—C11—C10—O4 = 99.3 (2)°.

### Experimental

The title compound was prepared following the literature methods (Dmowski & Piasecka-Macieiewska, 1998) with a slight modification. The compound crystallized from the oily reaction mixture that remained after acidification of the potassium benzoate salt with concentrated HCl, extraction of the organic components with toluene, drying of the organic fraction with magnesium sulfate and concentration by rotary evaporation.

### Refinement

H atoms were placed in geometrically idealized positions with C—H = 0.93 Å and O—H = 0.82 Å and constrained to ride on the parent atom with *U*_iso_(H) = 1.2 *U*_eq_(C) or 1.5 *U*_eq_(O). The trifluoromethyl groups belonging to C17 and C18 were modeled with a two-site disorder of the F atoms with refined site occupancy factors of 0.569 (5):0.431 (5) and 0.689 (5):0.311 (5), respectively.

### Crystal data

**Table:**

C_9_H_4_F_6_O_2_	*F*(000) = 1024
*M**_r_* = 258.12	*D*_x_ = 1.738 Mg m^−^^3^
Monoclinic, *P*2_1_/*c*	Mo *K*α radiation, λ = 0.71073 Å
Hall symbol: -P 2ybc	Cell parameters from 6970 reflections
*a* = 10.873 (2) Å	θ = 2.3–28.0°
*b* = 15.755 (3) Å	µ = 0.20 mm^−^^1^
*c* = 11.561 (2) Å	*T* = 296 K
β = 94.961 (2)°	Block, colorless
*V* = 1973.0 (6) Å^3^	0.39 × 0.31 × 0.26 mm
*Z* = 8	

### Data collection

**Table:**

Bruker APEXII CCD diffractometer	3438 independent reflections
Radiation source: fine-focus sealed tube	2889 reflections with *I* > 2σ(*I*)
graphite	*R*_int_ = 0.021
φ and ω scans	θ_max_ = 25.0°, θ_min_ = 2.2°
Absorption correction: multi-scan (*SADABS*; Bruker, 2008)	*h* = −12→12
*T*_min_ = 0.834, *T*_max_ = 0.951	*k* = −18→18
12904 measured reflections	*l* = −13→11

### Refinement

**Table:**

Refinement on *F*^2^	Primary atom site location: structure-invariant direct methods
Least-squares matrix: full	Secondary atom site location: difference Fourier map
*R*[*F*^2^ > 2σ(*F*^2^)] = 0.039	Hydrogen site location: inferred from neighbouring sites
*wR*(*F*^2^) = 0.112	H-atom parameters constrained
*S* = 1.02	*w* = 1/[σ^2^(*F*_o_^2^) + (0.058*P*)^2^ + 0.5224*P*] where *P* = (*F*_o_^2^ + 2*F*_c_^2^)/3
3438 reflections	(Δ/σ)_max_ < 0.001
331 parameters	Δρ_max_ = 0.21 e Å^−^^3^
0 restraints	Δρ_min_ = −0.23 e Å^−^^3^

### Special details

**Table:**

Geometry. All e.s.d.'s (except the e.s.d. in the dihedral angle between two l.s. planes) are estimated using the full covariance matrix. The cell e.s.d.'s are taken into account individually in the estimation of e.s.d.'s in distances, angles and torsion angles; correlations between e.s.d.'s in cell parameters are only used when they are defined by crystal symmetry. An approximate (isotropic) treatment of cell e.s.d.'s is used for estimating e.s.d.'s involving l.s. planes.
Refinement. Refinement of *F*^2^ against ALL reflections. The weighted *R*-factor *wR* and goodness of fit *S* are based on *F*^2^, conventional *R*-factors *R* are based on *F*, with *F* set to zero for negative *F*^2^. The threshold expression of *F*^2^ > σ(*F*^2^) is used only for calculating *R*-factors(gt) *etc*. and is not relevant to the choice of reflections for refinement. *R*-factors based on *F*^2^ are statistically about twice as large as those based on *F*, and *R*- factors based on ALL data will be even larger.

### Fractional atomic coordinates and isotropic or equivalent isotropic displacement parameters (Å^2^)

**Table:**

	*x*	*y*	*z*	*U*_iso_*/*U*_eq_	Occ. (<1)
C1	0.25273 (16)	0.14562 (11)	0.00186 (14)	0.0532 (4)	
C2	0.34011 (15)	0.08652 (10)	0.07069 (14)	0.0490 (4)	
C3	0.43893 (16)	0.11687 (12)	0.14433 (16)	0.0564 (4)	
C4	0.51596 (17)	0.06028 (14)	0.20807 (17)	0.0666 (5)	
H4A	0.5807	0.0808	0.2581	0.080*	
C5	0.49740 (19)	−0.02557 (14)	0.19783 (18)	0.0689 (5)	
H5A	0.5498	−0.0629	0.2405	0.083*	
C6	0.40178 (18)	−0.05640 (12)	0.12489 (16)	0.0614 (5)	
H6A	0.3900	−0.1147	0.1175	0.074*	
C7	0.32257 (15)	−0.00131 (10)	0.06209 (14)	0.0508 (4)	
C8	0.46743 (19)	0.20978 (14)	0.1562 (2)	0.0756 (6)	
F1	0.5244 (2)	0.22865 (11)	0.25885 (18)	0.1361 (7)	
F2	0.53907 (15)	0.23619 (9)	0.07602 (19)	0.1232 (7)	
F3	0.36850 (12)	0.25931 (8)	0.14440 (14)	0.0893 (4)	
C9	0.21830 (18)	−0.03876 (12)	−0.01534 (17)	0.0632 (5)	
F4	0.11518 (12)	−0.04286 (10)	0.03733 (13)	0.0975 (4)	
F5	0.19338 (14)	0.00483 (8)	−0.11254 (11)	0.0911 (4)	
F6	0.24344 (13)	−0.11707 (8)	−0.04877 (12)	0.0906 (4)	
C10	0.10257 (17)	0.15284 (11)	0.30263 (15)	0.0545 (4)	
C11	0.07034 (16)	0.13002 (10)	0.42282 (14)	0.0514 (4)	
C12	−0.04150 (17)	0.15569 (11)	0.46310 (15)	0.0561 (4)	
C13	−0.0643 (2)	0.14110 (13)	0.57757 (18)	0.0711 (5)	
H13A	−0.1380	0.1593	0.6045	0.085*	
C14	0.0220 (2)	0.09977 (15)	0.65140 (18)	0.0801 (6)	
H14A	0.0066	0.0906	0.7283	0.096*	
C15	0.1301 (2)	0.07212 (14)	0.61252 (17)	0.0730 (6)	
H15A	0.1869	0.0430	0.6626	0.088*	
C16	0.15558 (18)	0.08709 (12)	0.49899 (16)	0.0603 (5)	
O1	0.14736 (11)	0.15753 (8)	0.02411 (10)	0.0606 (3)	
O2	0.30404 (14)	0.18036 (9)	−0.08444 (12)	0.0751 (4)	
H2A	0.2549	0.2129	−0.1192	0.113*	
O3	0.05615 (13)	0.10397 (9)	0.22093 (11)	0.0684 (4)	
H3A	0.0818	0.1182	0.1592	0.103*	
O4	0.16936 (19)	0.21110 (11)	0.28586 (13)	0.0999 (6)	
C17	−0.13950 (10)	0.19782 (8)	0.38373 (10)	0.0726 (5)	
F7A	−0.09321 (12)	0.25255 (8)	0.31618 (10)	0.1010 (13)	0.569 (5)
F8A	−0.19164 (10)	0.13805 (10)	0.31178 (10)	0.0901 (11)	0.569 (5)
F9A	−0.22489 (10)	0.23045 (8)	0.43893 (12)	0.139 (2)	0.569 (5)
F7B	−0.17175 (10)	0.16347 (9)	0.28614 (11)	0.144 (3)	0.431 (5)
F9B	−0.10986 (11)	0.28028 (10)	0.35381 (9)	0.1164 (19)	0.431 (5)
F8B	−0.24254 (11)	0.21614 (8)	0.43317 (12)	0.0970 (19)	0.431 (5)
C18	0.27540 (10)	0.05569 (8)	0.46034 (11)	0.0751 (6)	
F10A	0.26369 (10)	0.01894 (8)	0.35789 (13)	0.0877 (11)	0.689 (5)
F11A	0.36230 (10)	0.11205 (10)	0.46284 (11)	0.1163 (14)	0.689 (5)
F12A	0.32020 (9)	−0.01015 (9)	0.52898 (13)	0.1142 (11)	0.689 (5)
F10B	0.33236 (9)	0.12165 (10)	0.40107 (11)	0.108 (2)	0.311 (5)
F12B	0.35672 (10)	0.03614 (8)	0.54011 (13)	0.125 (3)	0.311 (5)
F11B	0.26173 (10)	0.00490 (9)	0.37781 (12)	0.154 (5)	0.311 (5)

### Atomic displacement parameters (Å^2^)

**Table:**

	*U*^11^	*U*^22^	*U*^33^	*U*^12^	*U*^13^	*U*^23^
C1	0.0618 (10)	0.0542 (9)	0.0442 (9)	0.0104 (7)	0.0085 (7)	0.0040 (7)
C2	0.0497 (8)	0.0552 (9)	0.0428 (8)	0.0073 (7)	0.0077 (7)	0.0041 (7)
C3	0.0508 (9)	0.0623 (10)	0.0566 (10)	0.0011 (8)	0.0089 (8)	−0.0018 (8)
C4	0.0507 (10)	0.0864 (14)	0.0607 (11)	0.0064 (9)	−0.0062 (8)	−0.0020 (10)
C5	0.0631 (11)	0.0776 (13)	0.0641 (12)	0.0199 (10)	−0.0061 (9)	0.0105 (10)
C6	0.0689 (11)	0.0567 (10)	0.0582 (11)	0.0113 (8)	0.0025 (9)	0.0077 (8)
C7	0.0535 (9)	0.0551 (9)	0.0436 (9)	0.0056 (7)	0.0044 (7)	0.0031 (7)
C8	0.0608 (11)	0.0702 (12)	0.0965 (16)	−0.0044 (10)	0.0112 (11)	−0.0113 (11)
F1	0.1536 (16)	0.0987 (11)	0.1447 (15)	−0.0163 (10)	−0.0518 (13)	−0.0361 (10)
F2	0.1085 (11)	0.0773 (9)	0.1956 (19)	−0.0172 (8)	0.0813 (12)	−0.0035 (10)
F3	0.0828 (8)	0.0627 (7)	0.1245 (12)	0.0037 (6)	0.0221 (8)	−0.0124 (7)
C9	0.0672 (11)	0.0597 (11)	0.0615 (11)	0.0030 (9)	−0.0013 (9)	0.0003 (9)
F4	0.0628 (7)	0.1270 (12)	0.1024 (10)	−0.0164 (7)	0.0050 (7)	−0.0162 (9)
F5	0.1201 (11)	0.0832 (8)	0.0628 (7)	−0.0013 (7)	−0.0338 (7)	0.0035 (6)
F6	0.1079 (10)	0.0632 (7)	0.0972 (10)	0.0013 (6)	−0.0118 (8)	−0.0186 (6)
C10	0.0609 (10)	0.0574 (10)	0.0454 (9)	−0.0107 (8)	0.0059 (7)	−0.0075 (7)
C11	0.0640 (10)	0.0495 (9)	0.0408 (8)	−0.0136 (7)	0.0046 (7)	−0.0079 (7)
C12	0.0679 (11)	0.0526 (9)	0.0486 (9)	−0.0112 (8)	0.0089 (8)	−0.0117 (7)
C13	0.0839 (13)	0.0731 (12)	0.0591 (12)	−0.0128 (10)	0.0231 (10)	−0.0126 (10)
C14	0.1122 (18)	0.0860 (15)	0.0440 (10)	−0.0135 (13)	0.0171 (11)	0.0010 (10)
C15	0.0927 (15)	0.0781 (13)	0.0473 (10)	−0.0043 (11)	0.0009 (10)	0.0031 (9)
C16	0.0698 (11)	0.0606 (10)	0.0499 (10)	−0.0094 (9)	0.0017 (8)	−0.0042 (8)
O1	0.0575 (7)	0.0753 (8)	0.0490 (7)	0.0156 (6)	0.0041 (5)	0.0077 (6)
O2	0.0858 (9)	0.0756 (9)	0.0677 (8)	0.0301 (7)	0.0294 (7)	0.0303 (7)
O3	0.0850 (9)	0.0783 (9)	0.0420 (6)	−0.0259 (7)	0.0064 (6)	−0.0103 (6)
O4	0.1505 (15)	0.0963 (11)	0.0575 (9)	−0.0706 (11)	0.0357 (9)	−0.0223 (8)
C17	0.0723 (13)	0.0783 (14)	0.0675 (13)	0.0023 (11)	0.0087 (10)	−0.0092 (11)
F7A	0.115 (2)	0.088 (2)	0.099 (2)	0.0095 (16)	0.0008 (17)	0.0347 (18)
F8A	0.0718 (17)	0.0977 (19)	0.095 (2)	−0.0026 (14)	−0.0251 (14)	−0.0169 (16)
F9A	0.153 (4)	0.144 (3)	0.121 (4)	0.087 (3)	0.022 (3)	−0.030 (3)
F7B	0.156 (5)	0.187 (5)	0.079 (3)	0.090 (4)	−0.040 (3)	−0.064 (3)
F9B	0.100 (3)	0.097 (3)	0.151 (4)	0.009 (2)	0.005 (3)	0.039 (3)
F8B	0.055 (2)	0.146 (4)	0.092 (4)	0.006 (3)	0.018 (2)	0.007 (3)
C18	0.0736 (13)	0.0807 (15)	0.0699 (14)	−0.0014 (11)	0.0001 (11)	−0.0030 (11)
F10A	0.082 (2)	0.116 (2)	0.0655 (15)	0.0210 (16)	0.0119 (13)	−0.0152 (16)
F11A	0.0730 (14)	0.115 (2)	0.161 (3)	−0.0229 (14)	0.0114 (17)	−0.033 (2)
F12A	0.1041 (18)	0.130 (2)	0.107 (2)	0.0381 (17)	−0.0002 (15)	0.0232 (16)
F10B	0.076 (3)	0.109 (4)	0.146 (6)	0.006 (3)	0.039 (3)	0.029 (4)
F12B	0.084 (3)	0.204 (9)	0.081 (4)	0.039 (5)	−0.020 (3)	0.023 (4)
F11B	0.099 (7)	0.145 (7)	0.222 (11)	−0.022 (5)	0.038 (6)	−0.109 (7)

### Geometric parameters (Å, °)

**Table:**

C1—O1	1.210 (2)	C11—C12	1.399 (3)
C1—O2	1.305 (2)	C12—C13	1.387 (3)
C1—C2	1.507 (2)	C12—C17	1.500 (2)
C2—C3	1.396 (2)	C13—C14	1.376 (3)
C2—C7	1.399 (2)	C13—H13A	0.930
C3—C4	1.390 (3)	C14—C15	1.365 (3)
C3—C8	1.500 (3)	C14—H14A	0.930
C4—C5	1.371 (3)	C15—C16	1.385 (3)
C4—H4A	0.930	C15—H15A	0.930
C5—C6	1.370 (3)	C16—C18	1.498 (2)
C5—H5A	0.930	O2—H2A	0.820
C6—C7	1.383 (2)	O3—H3A	0.820
C6—H6A	0.930	C17—F7B	1.2729 (11)
C7—C9	1.503 (3)	C17—F9A	1.2791 (11)
C8—F1	1.324 (3)	C17—F7A	1.2941 (11)
C8—F3	1.326 (3)	C17—F8B	1.3326 (12)
C8—F2	1.328 (3)	C17—F8A	1.3485 (12)
C9—F4	1.323 (2)	C17—F9B	1.3895 (12)
C9—F5	1.325 (2)	C18—F11B	1.2443 (10)
C9—F6	1.328 (2)	C18—F12B	1.2593 (11)
C10—O4	1.197 (2)	C18—F11A	1.2952 (11)
C10—O3	1.287 (2)	C18—F10A	1.3146 (11)
C10—C11	1.506 (2)	C18—F12A	1.3697 (11)
C11—C16	1.397 (3)	C18—F10B	1.4165 (12)
			
O1—C1—O2	124.93 (16)	C13—C12—C17	118.76 (16)
O1—C1—C2	123.28 (15)	C11—C12—C17	121.09 (14)
O2—C1—C2	111.78 (14)	C14—C13—C12	120.1 (2)
C3—C2—C7	118.36 (15)	C14—C13—H13A	119.9
C3—C2—C1	121.80 (15)	C12—C13—H13A	119.9
C7—C2—C1	119.84 (15)	C15—C14—C13	120.45 (19)
C4—C3—C2	120.00 (17)	C15—C14—H14A	119.8
C4—C3—C8	117.85 (18)	C13—C14—H14A	119.8
C2—C3—C8	122.14 (17)	C14—C15—C16	120.4 (2)
C5—C4—C3	120.63 (18)	C14—C15—H15A	119.8
C5—C4—H4A	119.7	C16—C15—H15A	119.8
C3—C4—H4A	119.7	C15—C16—C11	120.24 (19)
C6—C5—C4	120.08 (17)	C15—C16—C18	118.50 (17)
C6—C5—H5A	120.0	C11—C16—C18	121.25 (15)
C4—C5—H5A	120.0	C1—O2—H2A	109.5
C5—C6—C7	120.36 (18)	C10—O3—H3A	109.5
C5—C6—H6A	119.8	F9A—C17—F7A	111.7
C7—C6—H6A	119.8	F7B—C17—F8B	107.2
C6—C7—C2	120.55 (16)	F9A—C17—F8A	107.7
C6—C7—C9	118.00 (16)	F7A—C17—F8A	104.9
C2—C7—C9	121.45 (15)	F7B—C17—F9B	103.2
F1—C8—F3	105.80 (19)	F8B—C17—F9B	97.2
F1—C8—F2	107.3 (2)	F7B—C17—C12	118.73 (8)
F3—C8—F2	105.3 (2)	F9A—C17—C12	112.38 (8)
F1—C8—C3	112.2 (2)	F7A—C17—C12	111.79 (8)
F3—C8—C3	113.92 (17)	F8B—C17—C12	114.37 (8)
F2—C8—C3	111.77 (18)	F8A—C17—C12	107.87 (8)
F4—C9—F5	107.23 (17)	F9B—C17—C12	113.47 (8)
F4—C9—F6	107.01 (17)	F11B—C18—F12B	115.7
F5—C9—F6	105.48 (16)	F11A—C18—F10A	109.6
F4—C9—C7	111.76 (16)	F11A—C18—F12A	106.5
F5—C9—C7	112.95 (16)	F10A—C18—F12A	101.0
F6—C9—C7	111.98 (16)	F11B—C18—F10B	97.4
O4—C10—O3	123.01 (17)	F12B—C18—F10B	103.0
O4—C10—C11	121.71 (15)	F11B—C18—C16	113.07 (8)
O3—C10—C11	115.25 (15)	F12B—C18—C16	115.85 (8)
C16—C11—C12	118.60 (16)	F11A—C18—C16	114.75 (8)
C16—C11—C10	120.19 (16)	F10A—C18—C16	113.29 (8)
C12—C11—C10	121.10 (16)	F12A—C18—C16	110.63 (9)
C13—C12—C11	120.14 (18)	F10B—C18—C16	109.27 (9)
			
C7—C2—C1—O1	71.5 (2)	C12—C11—C10—O4	99.3 (2)

### Hydrogen-bond geometry (Å, °)

**Table:**

*D*—H···*A*	*D*—H	H···*A*	*D*···*A*	*D*—H···*A*
O2—H2A···O4^i^	0.82	1.82	2.6340 (19)	169
O3—H3A···O1	0.82	1.88	2.6951 (18)	176

Symmetry codes: (i) *x*, −*y*+1/2, *z*−1/2.
